# Integrating Smoking Cessation Treatment Into Web-Based Usual Psychological Care for People With Common Mental Illness: Feasibility Randomized Controlled Trial (ESCAPE Digital)

**DOI:** 10.2196/78424

**Published:** 2025-12-05

**Authors:** Gemma Taylor, Pamela Jacobsen, Anna Blackwell, Shadi Daryan, Deborah Roy, Daniel Duffy, Garrett Hisler, Katherine Sawyer, Ben Ainsworth, Douglas Hiscock, Sophia Papadakis, Jamie Brown, Marcus Munafò, Paul Aveyard

**Affiliations:** 1 Department of Psychology University of Bath Bath United Kingdom; 2 School of Psychological Science University of Bristol Bristol United Kingdom; 3 Amwell Dublin Ireland; 4 Department of Psychology University of Southampton Southampton, null United Kingdom; 5 National Centre for Smoking Cessation and Training Dorchester United Kingdom; 6 Department of Behavioural Science and Health University College London London United Kingdom; 7 Nuffield Department of Primary Care Health Sciences University of Oxford Oxford United Kingdom

**Keywords:** smoking cessation, depression, anxiety, mental health, digital intervention, tobacco

## Abstract

**Background:**

Stopping smoking can improve mental health, with effect sizes similar to antidepressant treatment. Internet-based cognitive behavioral therapy (iCBT) provides evidence-based treatment for depression and anxiety, and digital interventions can support smoking cessation. However, combined digital smoking and mental health support is not currently available in UK health services.

**Objective:**

This feasibility trial aimed to investigate the acceptability and feasibility of a digital tailored smoking cessation intervention delivered alongside usual iCBT, and test trial procedures.

**Methods:**

The study design was a 2-armed, parallel groups, pragmatic, feasibility randomized controlled trial. Eligible participants were adult (18 years and older), regular smokers referred to iCBT from National Health Service Talking Therapies services in England. Participants were screened, consented, and randomized via a web-based platform and allocated to intervention (integrated smoking cessation support) or control (usual care) arms. Fully automated processes ensured allocation concealment. It was not possible to blind participants or clinicians to the behavioral intervention. Follow-ups via web-based questionnaires were completed at 3- and 6-months. Prespecified progression criteria, to determine the feasibility of the integrated intervention and trial procedures for a definitive trial, were enrolment of eligible clients (≥20%); recruitment to the target (≥80%); outcome data completeness (≥70%); and self-reported quit attempts in the intervention arm (≥8%).

**Results:**

A total of 309 participants were randomized: 154 to the intervention arm and 155 to the control arm. The proportion of eligible clients enrolled (309/1484, 21%) met the criteria for progression; however, the number randomized was below target (309/500, 62%). In the intervention arm, 18% (27/154) self-reported at least one quit attempt, which exceeded the progression criteria but was comparable to the control arm (32/155, 21%). High loss to follow-up meant data completeness was low (<30% across 6 key pilot clinical outcomes).

**Conclusions:**

Integrating smoking cessation within digital mental health treatment and using automated procedures to enroll and randomize participants appears feasible. Adjustments to site recruitment could improve participant recruitment; however, a large loss to follow-up undermines the feasibility of progression.

**Trial Registration:**

ISRCTN Registry ISRCTN10612149; https://www.isrctn.com/ISRCTN10612149

**International Registered Report Identifier (IRRID):**

RR2-10.1016/j.cct.2024.107541

## Introduction

Smoking is a serious threat to global public health, causing premature disease and death, as well as in nonsmokers exposed to second-hand smoke [1,2]. In the United Kingdom, there has been a decline in adult smoking prevalence from 29% in the 1990s to 12% in 2023 [3]. Although smoking prevalence has also decreased among people with mental illness, it remains much higher (depression and anxiety: 28%; long-term mental health condition: 34%; serious mental illness: 41%, in 2014/15) compared with the general population [4]. This disparity in smoking is a leading cause of a gap in life expectancy; people with mental illness die 10-20 years earlier compared with the general population [4]. People with mental illness are just as motivated to quit smoking as those without these difficulties [5]; however, they are 19% less likely to succeed [6].

Motivation to quit can be weakened by misconceptions that smoking tobacco can alleviate and even act as a coping mechanism for symptoms of depression and anxiety [7]; however, smoking may make mental illness worse [8], and quitting smoking may improve mental health. A recent Cochrane review showed that a reduction in anxiety and depression symptoms was associated with stopping smoking (standardized mean difference −0.28, 95% CI −0.43 to −0.13, –0.30, 95% CI −0.39 to −0.21), similar to the impact of taking antidepressant medication [9,10]. In addition, a systematic review of smoking cessation interventions for people with current or past depression found that adding psychosocial mood management, primarily cognitive behavioral therapy, enhanced quitting smoking compared with usual smoking cessation support [11]. There is also evidence that advising smokers with common mental illness that stopping smoking can improve their mental health, increases motivation to quit over advice that it can improve physical health alone [12]. Combining mental health and stop smoking support may improve not only abstinence outcomes but also mental health outcomes and motivation to quit.

In England, National Health Service (NHS) Talking Therapies, for anxiety and depression services (formerly known as Improving Access to Psychological Therapies [IAPT]), provide treatment for people with common mental illnesses. A recent study found that Talking Therapies provide an acceptable setting to offer integrated stop smoking support [12]. Talking Therapies services predominantly offer cognitive behavioral therapy in different formats, including face-to-face, groups, and digital (internet-based cognitive behavioral therapy [iCBT]), depending on need and availability. The latter provides an opportunity to reach a larger number of those in need whilst reducing time demand on clinicians [13], and guided iCBT yields equivalent outcomes to that of face-to-face support for depression and anxiety [13,14]. iCBT platforms such as SilverCloud Health by Amwell have been shown to improve depression and anxiety outcomes compared with waitlist controls and have more positive results compared with other low-intensity treatments (ie, guided self-help bibliotherapy, psychoeducational group therapy) [15,16].

A digital format may also be acceptable for delivering smoking cessation support. A Cochrane review found that tailored and interactive internet smoking cessation interventions, with (relative risk 1.69, 95% CI 1.30-2.18; n=2334) or without (relative risk 1.15, 95% CI 1.01-1.30; n=6786) behavioral support, can increase smoking cessation compared with nonactive controls [17]. A survey of adults in England found that almost half of smokers were interested in using a digital smoking cessation intervention, but very few had engaged with one [18]. Given the potential interest and reach of digital support, the modest effects of these interventions could translate to meaningful improvements to health, as well as cost savings for health services. Providing tailored digital smoking cessation support for people with mental illness offers an opportunity to reach a large number of people from a population with high smoking prevalence who are already accessing digital treatment.

The lack of combined smoking cessation and mental health treatment is a missed opportunity to optimize the support provided at a single point of engagement with health services. Helping people to understand the impact of smoking on their mental health and support cessation could improve physical and mental health outcomes. In this trial, we aimed to investigate (1) the acceptability and feasibility to patients and mental health staff of a tailored and integrated smoking cessation intervention delivered as part of usual web-based treatment via the SilverCloud iCBT platform; and (2) the acceptability and feasibility of trial procedures in terms of recruitment, randomization, retention, and data collection.

## Methods

### Study Design

The trial was a parallel groups 2-arm, web-based randomized controlled feasibility and pilot trial. The trial design and intervention were co-designed with patient and public involvement and engagement representatives with lived experience of smoking and mental health [19]. The trial was prospectively registered on the ISRCTN registry (02/02/2023; ISRCTN10612149).

### Ethical Considerations

Ethics approval was obtained from the NHS Health Research Authority and Wales Research Ethics Committee (Wales REC 6, 22/WA/0051, IRAS ID 304857). All participants gave written consent. Participants were reimbursed for their time (up to £30 [US $39.94] vouchers for follow-ups and £15 [US $19.98] for taking part in an optional follow-up interview).

### Recruitment

Participants were recruited from NHS Talking Therapies services (formerly known as IAPT) in England that provide iCBT for anxiety and depression via a digital platform – SilverCloud by Amwell. This is part of a stepped care model where clients are offered the lowest intensity treatment first.

All adult clients referred for supported digital (web-based) mental health treatment for depression, anxiety, or both (ie, self-guided iCBT with practitioner-led reviews) were screened for eligibility. Inclusion criteria were (1) being aged ≥18 years; (2) self-reported smoking cigarettes (including hand-rolled). There were no additional exclusion criteria, and we did not exclude people due to physical or mental health comorbidities. Participants were not required to consider stopping smoking to take part, as the intervention was designed to be suitable for people ambivalent about changing smoking behavior. Participants self-reported their gender, using the options: “man,” “woman,” “non-binary,” “I identify as [free text].” All eligible participants were provided with a digital participant information sheet, which could be downloaded and saved offline. Participants who agreed to take part provided written informed consent.

As all referrals into the trial came from NHS mental health services, and each participant’s individual identity was verified by their clinical team, this eliminated the risk of participants being able to enter the trial under multiple identities.

### Randomization and Masking

Randomization was integrated into the automated digital recruitment process via the Qualtrics web-based platform. An algorithm in Qualtrics contains the “Mersenne Twister,” a standard general-purpose pseudorandom number generator to calculate a randomization sequence. Participants were allocated to the intervention or control group on a 1:1 allocation ratio. Allocation concealment was achieved as neither the researchers nor clinicians could anticipate or influence the next treatment allocation.

Participants were aware of their treatment allocation as they were informed of the treatment programs available to them as part of their digital therapy, and whether this included the smoking cessation program. This was also the case for the supporting clinician allocated to the service user. The risk of detection bias was low due to the design of the trial, in that all outcomes were self-reported and completed remotely by the participants, without contact with the research team.

### Procedures

#### Intervention Arm: Smoking Cessation Support + Talking Therapies Usual Care

All participants received usual care, that is, access to self-guided iCBT for anxiety or depression, which included approximately 6 reviews with a clinician over 6-12 weeks. Participants in the intervention arm also received smoking cessation support. Upon enrolment, participants received information about the impact that smoking could have on their mental health via email. This covered the role of nicotine withdrawal, the benefits of quitting, and information about where to find a smoking cessation program in their digital account, to motivate participants to engage with the program and the idea of quitting. Thereafter, smoking cessation was provided by the digital smoking cessation program, available alongside the participants’ mental health treatment program. The smoking cessation program was based on standard behavioral smoking cessation interventions [20,21] as currently delivered in NHS routine care, modified to be suitable for digital delivery and integrated within mental health support [22-24]. It consisted of 5 modules to help smokers move toward making a quit attempt as well as information about stop smoking aids (eg, nicotine replacement therapy and e-cigarettes). The intervention content was optimized in co-design workshops and think-aloud interviews, including people with lived experience of smoking and mental illness and clinicians with expertise in supporting iCBT interventions, aiming to make it acceptable and engaging [19]. The smoking cessation program was not tailored differently depending on participant’s primary reason for referral, or symptom profile at baseline.

#### Control Arm: Talking Therapies Usual Care

Participants received usual care. Participants in the control arm were signposted to NHS stop smoking information and support at the end of their participation in the trial.

Outcome assessments were conducted via a web-based platform at baseline, mid-therapy (5 assessment points at 2-weekly intervals after baseline), 3- and 6-month follow-up. If required, the research team sent email and phone prompts to participants to complete the follow-up assessments.

### Outcomes

#### Intervention Feasibility and Acceptability

A key early indicator of effectiveness is the proportion of people who report making a quit attempt, which can be measured in each arm of the trial. The outcome is recorded as the primary outcome measure on the ISRCTN Registry and is a key measure of intervention feasibility.

- Self-reported quit attempt: “Have you made a serious attempt to stop smoking (lasting at least 24 hours)?” (the specified timeframe was tailored to the assessment point [19]).
- Engagement with iCBT: Use of the smoking cessation (intervention arm only) and usual mental health treatment programs (eg, whether participants viewed any pages in the program).
- Self-reported use of smoking cessation medicine and e-cigarettes.
- Engagement with the Talking Therapies service (eg, number of attended appointments, treatment completion) to assess whether providing cessation support reduced engagement with standard treatment.
- Participant and clinician views of the intervention: (1) Modified version of the Stop Smoking Service Patient Satisfaction Survey (intervention arm only) [25]. (2) Qualitative interviews with participants in the intervention arm and clinicians that explored perspectives on the intervention, its delivery through SilverCloud alongside usual treatment, and the extent to which smoking cessation support impacted upon on mental health support and recovery.

#### Trial Feasibility and Acceptability

- Total recruitment: The number and rate of participants recruited relative to the target and as a proportion of eligible clients.
- Smoking cessation data completeness: The proportion of participants for whom abstinence data is returned at 3- and 6-month follow-up.
- Concordance between self-reported and biochemically validated abstinence: Biochemical verification of abstinence measured by salivary cotinine or anabasine.
- Saliva sample data completeness: The proportion of saliva test packs sent to participants that are returned and yield a verified measure of cotinine or anabasine.
- Mental health outcomes data completeness: The proportion of participants for whom depression, anxiety, and quality of life data are returned at 3- and 6-month follow-up.

#### Pilot Clinical Outcomes

These outcomes would be used to test the intervention in an effectiveness trial.

- Smoking abstinence: Abstinence was defined as not smoking more than 5 cigarettes during the abstinence period [9]: (4-weeks at 3 months; 12-week prolonged abstinence between 3 and 6 months; self-reported and biochemically validated if participants consented to returning a saliva sample via post for laboratory analysis [19]).
- Depression and anxiety: Patient Health Questionnaire-9 (PHQ-9) [26] scores; General Anxiety Disorder Questionnaire-7 (GAD-7) [27] scores.
- Quality of life: EQ Visual Analogue Scale from the EuroQuol 3D questionnaire.

### Statistical Analysis

We aimed to recruit 500 participants to assess the feasibility of recruitment and data collection. The sample size was considered achievable as recruitment procedures were automated, and approximately 29%-34% of digital IAPT service users are smokers [28]. Means, proportions, and a relevant measure of variance for each variable are reported for baseline measures. We used descriptive statistics to report the key feasibility and acceptability outcomes. We report percentages and the median for categorical variables, and the mean and SD for continuous variables. We report the RAG (red/amber/green) status of our prespecified progression criteria (Table 1), which indicates whether it would be feasible to progress to an effectiveness trial without modification. The rationale for the threshold for each progression criterion is detailed in full in the published trial protocol [19]. In brief, the “green” progression criteria for each metric were set in line with best practice for feasibility trials [29]. At least 80% of recruitment target, and no more than 30% missing outcome data are the consensus benchmarks for clinical trials, which are most likely to be able to successfully answer the research question they are designed to test [30]. The “green” threshold for the proportion of eligible patients who chose to enroll in the trial was set at 20%, as a lower rate than this would make reaching recruitment targets in a future larger trial unlikely to be achieved, given the baseline rates of adults who are referred to self-guided iCBT who are also smokers (estimated to be 25% based on previous studies [28]). The “green” threshold for self-reported quit attempts in the intervention group was set based on previous studies showing a likely range of 7%-25% [28].

**Table 1:** Feasibility trial progression criteria.

Progression criteria	Red (change to protocol needed) (%)	Amber (protocol changes may be needed) (%)	Green (no change to protocol needed) (%)
Proportion of eligible SilverCloud clients (ie, adult smokers) who enroll in trial	<15	15-19	≥20
Recruitment compared with target	<60	60-79	≥80
Data completeness of future trial outcomes (ie, self-reported abstinence status, depression, and anxiety scores)	<50	50-69	≥70
Behavioral marker of engagement with smoking cessation program: self-reported quit attempts in intervention group	<5	5-7	≥8

Statistical analyses were used to calculate estimates of pilot clinical outcomes; these should not be interpreted as tests of intervention effectiveness. Analyses were performed on an intention-to-treat (ITT) basis. Due to the automated digital randomization, it was not anticipated that participants could receive a different intervention than that intended. All analyses were conducted in R (version 4.2.0; R Foundation for Statistical Computing) or STATA (version 18.0; StataCorp). We report percentages and the median for categorical variables, and the mean and SD for continuous variables. We compared continuous mental health and quality of life outcome values between trial arms at 3- and 6-months using linear regression modeling, with adjustment for baseline values. We report coefficients and 95% CIs from regression models. We compared categorical abstinence outcome values between trial arms at 3- and 6-months using logistic regression. We report odds ratios and 95% CIs from regression models. We analyzed the complete case and imputed data. Those with missing smoking outcome data were assumed to be smoking [31].

### Protocol Amendments

Participant reimbursement was amended to increase engagement to include the addition of a £10 (US $13.32) voucher sent with the saliva sample kits and £15 (US $19.98) for completion of an interview (approval received 03/04/2024).

## Results

**Figure 1:**
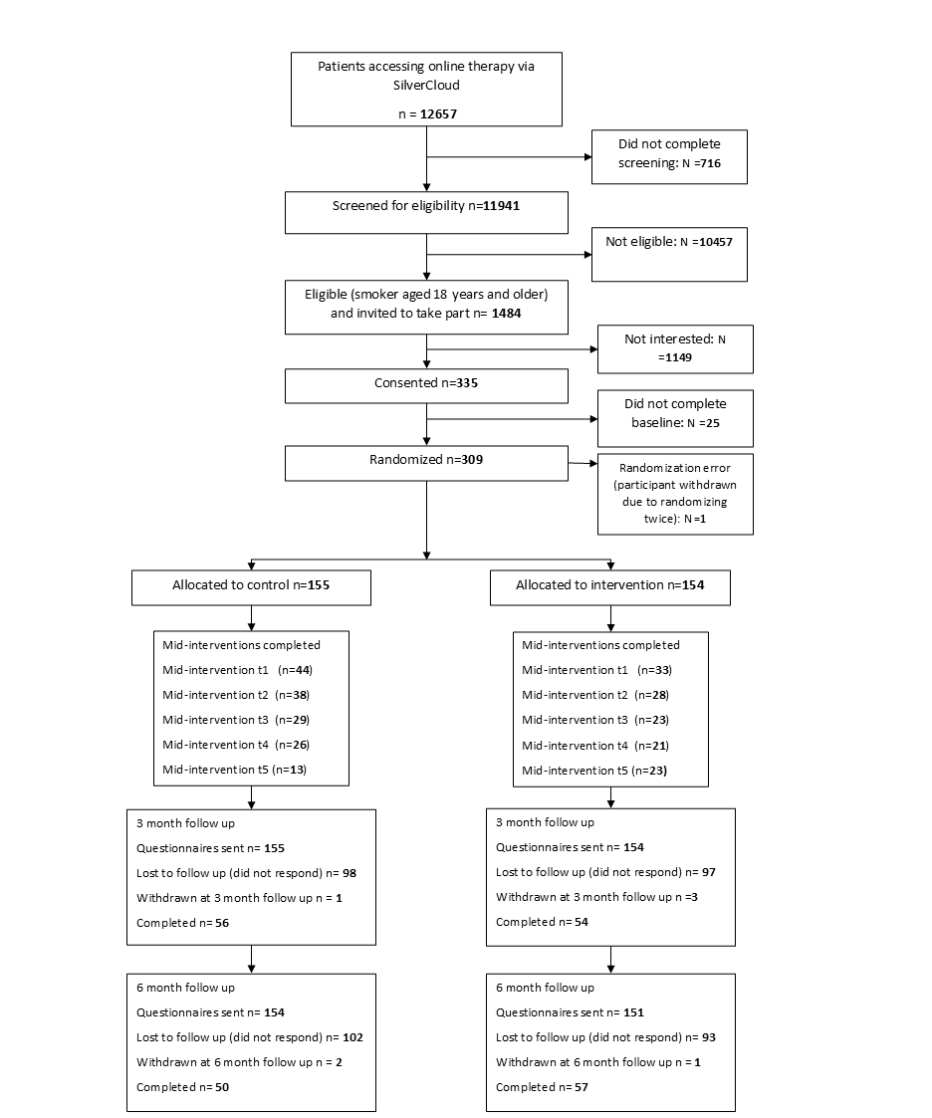
Flow through trial (CONSORT [Consolidated Standards of Reporting Trials] diagram).

Participants were recruited over a 14-month period from May 19, 2023, to June 28, 2024; final 6-month follow-ups were completed December 31, 2024. In total, 12,657 NHS Talking Therapies clients, from 17 services across 13 NHS trusts (ie, trial sites), were presented with information about the trial via their iCBT account (details for individual sites are provided in Table S1 in Multimedia Appendix 1). A total of 11,941 clients completed screening questions for inclusion and were excluded if they were not aged ≥18 years (n=1192) and not smokers (n=10,439). In addition, 1484 clients were eligible—which was 12% of the total supported SilverCloud population identified—and were invited to take part. A total of 341 (23%) of the eligible clients consented to take part; of these, 29 participants did not complete baseline assessments, and 1 participant was randomized twice and both records were excluded; therefore, 309 (21%) participants were randomized to the intervention (n=154) or control (n=155) arms. There was a large loss to follow-up in the intervention and control arms at 3-months (n=98 and n=97, respectively) and 6-months (n=93 and n=102, respectively). Furthermore, 60 (39%) intervention and 77 (50%) control participants reported whether they had made a quit attempt at any time point (assessed at 2, 4, 6, 8, 10 weeks, and 3 months post randomization). Partial or complete follow-up data were collected at 3 and 6 months from participants in the intervention (n=54, n=57, respectively) and control (n=56, n=50, respectively) arms (Figure 1 contains a CONSORT [Consolidated Standards of Reporting Trials] flow diagram; checklist provided in Multimedia Appendix 2). No serious adverse events were reported.

Participant baseline characteristics are reported in Table 2. The total sample consisted of 188 (61%) females, and the mean age was 37.7 years. The majority (95%) described their ethnicity as White, and 159 (51%) participants had completed school exams at the age of 18 years or older. The overall mean heaviness of the smoking index score was 2.4, which indicated low nicotine dependence. At the time of randomization, 15% of participants were using, and 63% had previously used, stop smoking medication or e-cigarettes. A total of 132 (43%) participants had made a quit attempt in the 12 months before starting the trial. PHQ-9 (mean 14.9, n=288) and GAD-7 (mean 13.1, n=288) scores indicate moderate (10-14) to moderately severe (15-19) depression and moderately severe (11-15) anxiety, respectively.

**Table 2:** Baseline characteristics of participants randomized to the intervention and control trial arms. Ethnicity categories are collapsed due to small numbers.

Measure			Intervention (n=154)		Control (n=155)
Age (years), mean (SD)			38.0 (12.0)		37.0 (11.0)
Number of cigarettes per day, mean (SD)			14.3 (7.8)		13.4 (7.9)
**Gender, n (%)**					
	Man	61 (39.6)		59 (38.1)	
	Woman	92 (59.7)		96 (61.9)	
	Other	2 (1.3)		0 (0.0)	
**Education, n (%)**					
	Higher education or equivalent	44 (28.6)		41 (26.5)	
	A level or equivalent	34 (22.1)		39 (25.2)	
	GCSE^a^ grade A*‐C or equivalent	58 (37.7)		48 (31.0)	
	Qualifications at level ≤1	4 (2.6)		12 (7.7)	
	Other qualifications: level unknown	4 (2.6)		6 (3.9)	
	No qualifications	10 (6.5)		9 (5.8)	
**Ethnicity, n (%)**					
	White	148 (96.1)		146 (94.2)	
	Other	6 (3.9)		9 (5.8)	
**Heaviness of Smoking Index, n (%)**					
	Low (0-2)	67 (43.5)		75 (48.4)	
	Medium (3-4)	73 (47.4)		70 (45.2)	
	High (5-6)	14 (9.1)		10 (6.5)	
**Quit attempt (≥24 h in past 12 months), n (%)**					
	Yes	68 (44.2)		63 (40.6)	
	No	86 (55.8)		92 (59.4)	
**Current use of stop smoking medication/e-cigarettes, n (%)**					
	Yes	25 (16.2)		21 (13.5)	
	No	129 (83.8)		134 (86.5)	
**Previous use of stop smoking medication/e-cigarettes, n (%)**					
	Yes	103 (66.9)		90 (58.1)	
	No	51 (33.1)		65 (41.9)	
**Primary reason for referral, n (%)**					
	Depressive disorders	81 (52.6)		80 (51.6)	
	Anxiety disorders	61 (39.6)		66 (42.6)	
	Other	5 (3.2)		1 (0.6)	
**Co-morbidities, n/N (%)**					
	Asthma	16/150 (10.7)		11/154 (7.1)	
	Diabetes	7/150 (4.7)		9/154 (5.8)	
	Hypertension	5/150 (3.3)		3/154 (1.9)	
PHQ-9^b^, mean (SD)			15.2 (5.7)		14.5 (5.7)
GAD-7^c^, mean (SD)			12.9 (4.8)		13.3 (4.8)
EQ-VAS^d^, mean (SD)			55.7 (21.0)		57.7 (19.3)

^a^GCSE: General Certificate of Secondary Education.

^b^PHQ-9: Patient Health Questionnaire-9.

^c^GAD-7: General Anxiety Disorder Questionnaire-7.

^d^EQ-VAS: EuroQuol Visual Analogue Scale.

The proportion of eligible clients (ie, adult smokers) who enrolled in the trial (21%) met our criteria for progression to a full-scale trial (Table 3). The proportion of participants who consented (68%) or were randomized (62%) to the trial relative to the target sample size (n=500) was within the amber status for progression. Data completeness of the pilot clinical outcomes was 27%-28% at 3- and 6-months follow-up, which was below the proportion required to support progression. Self-reported quit attempt in the intervention arm was one behavioral marker of engagement with the smoking cessation program; 27 (18%) participants reported at least one quit attempt, which exceeded the criteria for progression. In the control arm, 32 (21%) participants reported making a quit attempt.

**Table 3:** Trial and intervention feasibility based on the prespecified progression criteria.

Progression criteria		Result, n/N (%)	Progression status
Proportion of eligible clients who enroll in trial		309/1484 (21)	Green (no change to protocol needed)
Recruitment compared with target		309/500 (62)	Amber (protocol changes may be needed)
**Data completeness of pilot clinical outcomes**		<30	Red (change to protocol needed)
	Abstinence reported at 3 months	89/309 (29)	
	Abstinence reported at 6 months	87/309 (28)	
	Mental health reported at 3 months	89/309 (29)	
	Mental health reported at 6 months	83/309 (27)	
	Quality of life reported at 3 months	84/309 (28)	
	Quality of life reported at 6 months	83/309 (27)	
Self-reported quit attempts in the intervention group (at least one reported quit attempt).		27/154 (18)	Green (no change to protocol needed)

In the intervention arm, 33 (21%) participants engaged with the smoking cessation program (ie, viewed at least one page) and 16 (10%) participants viewed more than one module (5 modules in total). Most participants in the intervention (134/154, 87%) and control 135/155, 87%) arms engaged with their mental health program. Engagement with mental health services was similar across the intervention and control arms: mean number of attended appointments (2.47 and 2.69, respectively); the proportion who were recorded as completing treatment (24% and 26%, respectively) or “did not attend” (45% and 41%, respectively; Tables S2 and S3 in Multimedia Appendix 1 provide details).

Trial feasibility was also assessed by the completeness of outcome data collected at 3- and 6-month follow-ups. At 3- and 6-months, approximately a quarter of participants reported abstinence status (29% and 28%) and provided depression (PHQ-9: 29% and 27%), anxiety (GAD-7: 29% and 27%), or quality of life (EQ-VAS: 28% and 27%) scores (Table S4 in Multimedia Appendix 1). Manual reminders to complete follow-ups were sent to the majority of participants at 3-months (274/309, 89%, mean 3.3) and 6-months (276/309, 89%, mean 2.6). To increase key pilot clinical outcome data collection, smoking abstinence data were recorded directly by the research team during phone reminders for some participants at 3- (12/150, 8%) and 6-months (12/150, 8%).

Thirty-one participants at 3 months reported abstinence, 24 participants provided a postal address and were sent saliva sample kits, and 14 kits were returned to the laboratory for testing. At 6 months, 35 participants reported smoking abstinence, 28 participants provided a postal address, and 11 kits were returned to the laboratory. A total of 52 saliva kits were sent, and 25 kits arrived for testing (48%). At 3 months, 9 of the 14 samples were tested for cotinine only, and 5 were tested for anabasine; 12 tests validated abstinence (86%). At 6 months, 6 of the 11 samples were tested for cotinine only and 5 were tested for anabasine; 7 tests validated abstinence (64%).

Follow-up completion rates were similar across outcomes for the intervention and control arms. A total of 79 (26%) participants completed the satisfaction survey at 6-month follow-up. Responses demonstrated that they were satisfied with the information provided and questionnaires used and would take part in a similar study again (Table S5 in Multimedia Appendix 1 provides details).

Pilot clinical outcomes were analyzed using complete case data and, on an ITT basis. Using complete case data at 3-month and 6-month follow-ups, there was no strong evidence for differences in depression, anxiety, and quality of life scores between trial arms, and this remained consistent after imputing missing outcome data (Table 4). Using complete case data, 9/39 in the intervention arm and 3/50 in the control arm achieved bio-validated abstinence at 3-months follow-up (odds ratio [OR] 4.7, 95% CI 1.2-18.8), and 3/17 in the intervention and 4/13 in the control at 6-months (OR 0.5, 95% CI 0.9-2.7). Using an ITT analysis where participants with missing smoking cessation outcome data were assumed to be continuing smokers, 9/154 in the intervention arm and 3/155 in the control arm achieved bio-validated abstinence at 3-months follow-up (OR 3.1, 95% CI 0.8-11.9), and 3/154 in the intervention and 4/155 in the control at 6-months (OR 0.8, 95% CI 0.2-3.4; Table 4).

**Table 4:** Pilot clinical outcomes at 3 and 6 months for the effect of random allocation to the treatment arm on clinical outcomes. Beta coefficients or odds ratios derived from linear or logistic regression models and 95% CIs are presented. Estimates derived from complete case or imputed data.

				Intervention		Control		Coefficient (95% CI)
**3-month follow-up**								
	**Self-reported abstinence, n/N**							
		Complete case (N=89)	15/39		16/50		1.3 (0.6 to 3.2)	
		ITT^a^ (N=309)	15/154		16/155		0.9 (0.5 to 2.0)	
	**Bio validated abstinence, n/N**							
		Complete case (N=89)	9/39		3/50		4.7 (1.2 to 18.8)	
		ITT (N=309)	9/154		3/155		3.1 (0.8 to 11.9)	
	**PHQ-9^b^ (n=89), mean (SD)**							
		Complete case (N=81)	17.3 (5.7)		19.9 (7.6)		–2.0 (–4.7 to 0.8)	
		ITT (N=309)	19.0 (6.8)		20.0 (7.4)		–1.7 (–4.4 to 1.1)	
	**GAD-7^c^ (n=86), mean (SD)**							
		Complete case (N=78)	14.1 (5.0)		15.8 (6.7)		–0.9 (–3.5 to 1.7)	
		ITT (N=309)	16.5 (5.9)		16.1 (6.3)		–0.2 (–2.4 to 1.9)	
	**EQ-VAS (n=87), mean (SD)**							
		Complete case (N=87)	60.5 (20.7)		57.9 (20.4)		3.3 (–4.6 to 11.1)	
		ITT (N=309)	56.4 (22.0)		59.1 (22.6)		2.6 (–6.2 to 11.4)	
**6-month follow-up**								
	**Self-reported abstinence, n/N**							
		Complete case (N=87)	23/47		12/40		2.2 (0.9 to 5.4)	
		ITT (N=309)	23/154		12/155		2.1 (1.0 to 4.4)	
	**Bio validated abstinence, n/N**							
		Complete case (N=30)	3/17		4/13		0.5 (0.9 to 2.7)	
		ITT (N=309)	3/154		4/155		0.8 (0.2 to 3.4)	
	**PHQ-9 (n=89), mean (SD)**							
		Complete case (N=79)	17.5 (6.3)		18.3 (7.6)		–0.6 (–3.5 to 2.2)	
		ITT (N=309)	19.2 (6.7)		19.5 (7.4)		–0.2 (–3.7 to 3.4)	
	**GAD-7 (n=86), mean (SD)**							
		Complete case (N=78)	13.8 (4.8)		15.7 (6.9)		–1.5 (–3.9 to 1.0)	
		ITT (N=309)	17.4 (6.5)		16.7 (7.0)		–0.9 (–3.7 to 1.8)	
	**EQ-VAS (n=87), mean (SD)**							
		Complete case (N=82)	64.6 (19.4)		58.7 (15.5)		6.1 (–1.7 to 13.9)	
		ITT (N=309)	51.8 (24.3)		55.6 (20.6)		1.6 (–9.0 to 12.2)	

^a^ITT: intention-to-treat.

^b^PHQ-9: Patient Health Questionnaire-9.

^c^GAD-7: General Anxiety Disorder Questionnaire-7.

## Discussion

We recruited 309 participants to a pilot clinical trial of smoking cessation support within digital psychological therapy in NHS Talking Therapies services, for adult smokers receiving care for common mental health disorders. We found that the proportion of self-reported quit attempts in the intervention arm, which was a behavioral marker of engagement with the smoking cessation intervention, exceeded the preregistered progression criteria for success. However, a similar proportion of participants reported making a quit attempt in the control arm. The proportion of eligible clients (ie, adult smokers at the start of their digital mental health treatment) who enrolled in the trial met the progression criteria for success. In contrast, the overall number of participants recruited was below the target sample size and the threshold for progression. In addition, follow-up rates across trial arms were very low, which suggests that data collection for a definitive trial would not be feasible using the existing trial procedures.

The findings from the trial broadly support the feasibility of delivering a smoking cessation intervention alongside usual digital mental health treatment. It did not appear to affect engagement with mental health services: discharge status was similar across trial arms, as well as the proportion of participants who engaged with their usual digital mental health treatment program and the length and duration of program use. At the start of the trial, 15% of participants were currently using stop smoking aids, and 43% had made a quit attempt in the previous year. However, participants did not have to be interested in or motivated to quit to be eligible for the trial, and subsequent engagement with the smoking cessation intervention was self-determined. Approximately one out of 5 participants in the intervention arm engaged with the smoking cessation intervention. Furthermore, participants in the intervention arm reported an average satisfaction score of 3.9 out of 5, where “5=very satisfied” in terms of their overall experience of taking part in the trial. Participants also indicated a willingness to take part in a future trial, with an average score of 4 out of 5, where “5=very likely” (Table S5 in Multimedia Appendix 1). This indicates participants were satisfied with the trial and found the trial procedures and intervention acceptable. These findings should be interpreted with appropriate caution, however, as response bias effects could have led to participants who were more satisfied with the trial being more likely to complete the feedback questionnaire at 6-month follow-up.

A similar proportion of participants reported making at least one quit attempt between baseline and 3-month follow-up in the intervention and control arms. This feasibility trial did not aim to assess effectiveness; however, if quit attempts were similar across trial arms in a future trial, the intervention may still be effective if it increases smoking abstinence among those who try to quit, and there was uncertain evidence that the intervention improved self-reported cessation. The proportion of participants reporting a quit attempt was consistent with the proportion of people previously found to be planning to quit soon (ie, in the next 30 days; 19%) in a review of quitting intentions among people with mental illness [5]. The review found that a further 38% of people may consider quitting in the next few months, and that stop smoking support in this population may need to account for additional barriers to quitting and ensure that mental health practitioners understand why and how they can support people who smoke [5]. It is possible that the psychoeducational information provided to participants in the intervention arm in this trial was not sufficient to encourage them to view the smoking cessation program, and improvements could increase awareness of, and engagement with, the intervention. Options include signposting to the intervention and including psychoeducational information about smoking and mental health within the usual mental health treatment platform, which could be achieved without additional practitioner time, or could be facilitated through practitioner training to actively promote and review content with clients. This could increase motivation and confidence in quitting and support a shift from the contemplation to the planning stage of quitting.

This trial identified that recruitment and data collection for a full-scale trial would not be feasible using the methods used here. Recruitment of participants took longer and required more NHS trust sites than anticipated. This was mainly because the proportion of people smoking was approximately a third less than anticipated (12% vs 34%). Overall recruitment fell short of the target; however, most participants (70%) were randomized from just 4 of the 13 trusts. More targeted site recruitment may improve participant recruitment. The main concern regarding progression was the low proportion (<30%) of participants completing outcome assessments. Attrition bias is a general concern for internet-based smoking cessation intervention research; a Cochrane review found over a quarter of identified studies had loss to follow-up above 50% [17]. Participants in this trial were not required to be motivated to quit or be willing to make a serious quit attempt, and they were also recruited from a population of people accessing digital mental health services. The primary purpose for them in accessing the web-based mental health platform was to complete their planned mental health treatment and clinical assessments. Over 40% of participants had their mental health service discharge status recorded as “did not attend.” It is possible that the additional smoking cessation intervention and research questions related to their smoking were of low priority or additional burden. Including more direct contact with research staff and greater financial incentives may increase participant engagement in a future digital trial in which smoking cessation is a secondary intervention.

There are several strengths and limitations of this trial. National Institute for Health and Care Excellence guidelines, which provide evidence-based health care recommendations in the United Kingdom, recommend that people accessing mental health services should be supported to stop smoking at outpatient sites, but there is a lack of evidence regarding how to support this population effectively [21]. As far as we are aware, this was the first assessment of a digital smoking cessation intervention that was integrated into usual mental health treatment in the United Kingdom. The intervention was built on existing evidence-based smoking cessation support [20,22], tailored for use in mental health services [32], and co-designed by people with lived experience. This trial has demonstrated the feasibility of adapting smoking cessation support to a digital format and using the opportunity presented by digital solutions to deliver faster and more convenient care for the public. The trial also used automated procedures that embedded screening, consent, randomization, and baseline assessment within the mental health platform. This approach meant that the whole population of clients accessing digital treatment at participating mental health services was screened for eligibility, providing accurate estimates of adult smokers and the proportion willing to take part in a trial. These automated procedures also reduced the burden on participants and services. We found that these procedures were promising for enrolling and randomizing participants; however, the automated processes were not sufficient for collecting outcomes data. Participants were reimbursed for their time and effort in this trial (up to £30 [US $39.94] vouchers for follow-ups and £15 [US $19.98] for interview participation); however, this was only increased (from up to £10 [US $13.32] vouchers) toward the end of the trial. Including the higher reimbursement amount from the outset, as well as increasing the saliency of this in the patient information sheet, may help to increase future engagement. In addition, there were technical issues setting up the digital procedures and intervention that would need to be addressed in the future. The web-based mental health platform is set up according to individual service requirements; therefore, integrating the trial procedures involved bespoke technical alterations. Testing functionality required a lot of ongoing communication between different teams, and sometimes glitches were identified after recruitment had started, including issues with the availability of the intervention within some client accounts. A future trial would need to accommodate the time to recruit sites and test the web-based processes to maximize recruitment potential. Finally, given that depression and anxiety are highly comorbid [33-35], the ESCAPE Digital trial was designed to investigate the feasibility of offering smoking cessation as an add-on to standard care to people referred to iCBT, with either depression or anxiety. Patients accessing iCBT as part of NHS Talking Therapies services are given a choice of different treatment modules to access, including both depression and anxiety, in recognition of the large overlap between depression and anxiety, rather than these being distinct problem areas. We did, however, find in this study that it was possible to collect data on whether the primary reason for referral was for depression or anxiety, and also to collect disaggregated baseline data on depression and anxiety symptoms. In a future clinical efficacy trial, a potential hypothesis which could be explored further could relate to differences in treatment response trajectories for different subgroups based on baseline clinical characteristics (ie, mainly depression, mainly anxiety, mixed depression/anxiety).

In summary, it appears to be feasible and acceptable to integrate smoking cessation support within digital mental health treatment and use automated procedures to enroll and randomize participants into a trial. Recruiting a large sample of adult smokers accessing digital mental health treatment may be possible if efforts focus on identifying and engaging suitable mental health service sites (ie, that refer sufficiently large numbers of clients) and factor in substantial time to set up technical processes. However, large loss to follow-up undermines the feasibility of progression to an effectiveness trial, and further work is needed to address the threat of attrition bias in this and other digital smoking cessation intervention research. This issue may be particularly pertinent in a population accessing mental health services where engagement with interventions for depression or anxiety is the priority. Finally, there is strong evidence base for both the physical and mental health benefits of stopping smoking, including for people with mental health difficulties. Future research could robustly test the causal relationship between stopping smoking and subsequent improvement in mental health by collecting outcome data on mental health outcomes over longer follow-up periods for people who quit smoking. It would also be important to collect data on potential moderators and mediators of this effect, as other factors may be associated with whether people are more likely to successfully quit smoking and more likely to make a successful mental health recovery after intervention, such as social support, socioeconomic status, and trauma history.

## Data Availability

The study protocol and statistical analysis plan are available from the ISRCTN Registry. The datasets generated or analyzed during this study (deidentified participant data and data dictionary) are available from the University of Bath’s Research Data Archive [36]. Data access is restricted and will be made available to approved bona fide researchers after they have signed a data access agreement with the Research Data Services. Participants consented to the sharing of anonymized data at the start of the study.

## Appendix

Multimedia Appendix 1 Supplementary material.

Multimedia Appendix 2 CONSORT-eHEALTH (V 1.6.1) checklist.

## Abbreviations

CONSORT: Consolidated Standards of Reporting Trials
GAD-7: General Anxiety Disorder Questionnaire-7
IAPT: Improving Access to Psychological Therapies
iCBT: internet-based cognitive behavioral therapy
ITT: intention-to-treat
NHS: National Health Service
OR: odds ratio
PHQ-9: Patient Health Questionnaire-9
