# Supplementary material for: Integrating Smoking Cessation Treatment Into Web-Based Usual Psychological Care for People With Common Mental Illness: Feasibility Randomized Controlled Trial (ESCAPE Digital)
Source: JMIR Ment Health. 2025 Dec 5;12:e78424. doi: 10.2196/78424 (PMC12717507; doi:10.2196/78424)
Supplement: Multimedia Appendix 1 [file mental_v12i1e78424_app1.pdf]

IntEgrating Smoking Cessation treAtment into usual online Psychological care for people with common mEntal illness: An online randomised feasibility and pilot trial (ESCAPE Digital): Supplementary materials

Contents

Table S1 The number of supported SilverCloud clients, the number eligible for inclusion, the number consented and randomised at each trial site. 2

Table S2 Programme engagement and outcomes. 3

Table S3 Intervention acceptability at 3 months (intervention arm only): Intervention satisfaction questions. 4

Table S4 Trial feasibility outcomes: data completeness for clinical outcome data. 5

Trial and intervention acceptability 6

Table S5 Trial feasibility outcomes: participant satisfaction 7

Table S1 The number of supported SilverCloud clients, the number eligible for inclusion, the number consented and randomised at each trial site.

Site	Total clients	No. eligible	No. randomised	No. months recruiting	Recruitment rate (per month)
1	2413	278 (12%)	62	12	5
2	1680	268 (16%)	70	12	6
3	238	17 (7%)	5	9	0.6
4	767	114 (15%)	18	6	3
5	999	102 (10%)	23	10	2
6	90	12 (13%)	1	5	0.2
7	632	72 (11%)	9	11	0.8
8	1202	152 (13%)	33	8	4
9	764	77 (10%)	15	6	3
10	645	51 (8%)	13	5	3
11	2869	292 (10%)	51	7	7
12	192	24 (13%)	6	7	0.9
13	166	25 (15%)	4	3	1
**Total**	**12657**	**1484 (12%)**	**310**		

Table S2 Programme engagement and outcomes.

Question	Intervention (n = 154)	Control (n = 155)
Self-reported quit attempt - Yes	27/154 (17.5%)	32/155 (20.6%)
Self-reported quit attempt - No	33/154 (21.4%)	45/155 (29.0%)
Length of total support (days)	–	–
No. of logins	15.7 (18.6)	14.6 (17.6)
Duration per program (minutes)	155.6 (192.2)	161.6 (205.4)
Smoking cessation programme - Viewed at least one page	33 (21.4%)	N/A
Pages viewed (of 49)	17.9 (14.7)	N/A
Completion of smoking programme pages	590/1617 (36.5%) or 590/7546 (7.8%)	N/A
Mental health programme - No participants viewed	–	–
Mental Health modules: total pages viewed	134 pages	135 pages
Mental Health modules - Avg. pages viewed	43.6 (28.7)	43.1 (32.9)
Average time per session (minutes)	10.0	11.0
Completion rate (pages viewed)	5841 pages (43.6 (28.7))	5847 pages (43.3 (32.9))
Use of smoking aids - Yes	30/154 (19.5%)	22/155 (14.2%)
Use of smoking aids - No	52/154 (33.8%)	65/155 (41.9%)
Attended appointments	2.5 (2.1)	2.7 (2.4)
Missed appointments	0.3 (0.8)	0.4 (0.8)
Discharge status - Ongoing	1 (0.6%)	3 (1.9%)
Discharge status - DNA	67 (43.5%)	63 (40.6%)
Discharge status - Stepped up	24 (15.6%)	12 (7.7%)
Discharge status - Completed	36 (23.4%)	39 (25.2%)
Discharge status - Other	23 (14.9%)	22 (14.2%)

Note: smoking cessation programme included five modules (49 pages), there are 17 programmes, and 65 mental health modules

Table S3 Intervention acceptability at 3 months (intervention arm only): Intervention satisfaction questions.

Question	Response / Data (Mean, SD where applicable)
**How satisfied with overall programme**<br>(1 = very unsatisfied; 5 = very satisfied)<br>n = 30/154	3.3 (1.0)
**Would recommend smoking program to others?**<br>n = 30/154	Yes: 16 (10.4%)<br>No: 1 (0.6%)<br>Unsure: 13 (8.4%)
**Discussed smoking or quitting with clinician**<br>n = 31/154	No: 16 (44.0%)<br>Yes: 0 (0.0%)<br>Unsure: 9 (25.0%)<br>No contact: 11 (31.0%)
**How supportive staff have been**<br>(1 = very unsupportive; 5 = very supportive)<br>n = 9/154	4.1 (1.4)
**Helpfulness of information and advice in programme**<br>(1 = very unhelpful; 5 = very helpful)<br>n = 36/154	3.5 (1.0)
**Helpfulness of info about stop smoking aids**<br>(1 = very unhelpful; 5 = very helpful)<br>n = 35/154	2.3 (0.6)
**How did you get medication?**<br>n = 36/154	GP: 1 (0.6%)<br>Chemist: 3 (1.9%)<br>Other: 2 (1.3%)<br>Did not use meds: 30 (19.5%)
**Ease of getting smoking medication**<br>n = 6/154	No: 3 (1.9%)<br>Yes: 1 (0.6%)<br>Unsure: 2 (1.3%)

Table S4 Trial feasibility outcomes: data completeness for clinical outcome data.

**Smoking Abstinence and Mental Health Follow-Up Data**		
3-month follow-up	39 (25.3%)	48 (31.0%)
6-month follow-up	43 (27.9%)	39 (25.2%)
**Saliva Sample Kits Sent (% of those who provided address)**		
3-month follow-up	24	28
6-month follow-up	28	–
**Saliva Samples Returned (of abstinence cases reported)**		
Timepoint	Intervention	Control
3-month follow-up	10/15 (66.7%)	4/16 (25.0%)
6-month follow-up	5/23 (21.7%)	6/12 (50.0%)
**Abstinence Biochemically Validated (% of samples received at lab)**		
3-month follow-up	9/10 (90.0%)	3/4 (75.0%)
6-month follow-up	3/5 (60.0%)	4/6 (66.7%)
**Mental Health Data Complete (% of total group)**		
PHQ-9 – 3-month	40 (26.0%)	49 (31.6%)
PHQ-9 – 6-month	43 (27.9%)	40 (25.8%)
GAD-7 – 3-month	38 (24.7%)	48 (31.0%)
GAD-7 – 6-month	43 (27.9%)	39 (25.2%)
**Manual Reminders Sent (% of total group)**		
3-month follow-up	134/154 (87.0%)	140/155 (90.3%)
6-month follow-up	136/154 (88.3%)	140/155 (90.3%)
**Number of Reminders Sent per Participant (M = Mean)**		
3-month follow-up	3.3	3.0
6-month follow-up	2.5	2.7
**Pilot Clinical Outcomes Collected via Phone**		
3-month follow-up	5 (3.2%)	7 (4.5%)
6-month follow-up	5 (3.2%)	7 (4.5%)

Trial and intervention acceptability

Qualitative data

Acceptability of the intervention and procedures was also assessed using free-text responses (n=15) and qualitative interviews (n=3). A summary of the data includes both positive and negative views of the smoking cessation intervention and being part of the trial. More information about how to use the Saliva testing kit was mentioned but satisfaction with trial procedures, namely simplicity and ease of use of the survey instruments was prominent; something mirrored in answers to the trial satisfaction questions (see Table S5).

“Very easy. Yeah, it was really straightforward. And like I say, it wasn't time consuming or invasive. It was very, very well put together, I think”.

One participant found the smoking cessation content useful.

“I think it kind of flagged for me - when I smoke, and how often, which is quite, well, quite a lot, which was quite interesting, you don't really notice do you when you do smoke..”

In contrast, another of the three participants interviewed spoke of their preference for and benefits of using Zyban, a smoking medication, and did not find the smoking cessation online materials helpful or needed.

“Yeah. And they weren't really very useful at all, if I'm honest, especially considering I was on the Zyban that really kind of did it all.”

Table S5 Trial feasibility outcomes: participant satisfaction

**Reasons for Taking Part (n = 80/309)**		
**Reason**	**Intervention (n = 38/154)**	**Control (n = 42/155)**
To get stop smoking support	Yes = 19 (12.3%)<br>No = 23 (14.9%)	Yes = 19 (12.3%)<br>No = 23 (14.9%)
To get the shopping vouchers	Yes = 6 (3.9%)<br>No = 36 (23.4%)	Yes = 3 (1.9%)<br>No = 39 (25.2%)
To take part in research	Yes = 20 (13.0%)<br>No = 22 (14.3%)	Yes = 20 (13.0%)<br>No = 22 (14.3%)
To help my mental health	Yes = 23 (14.9%)<br>No = 15 (9.7%)	Yes = 22 (14.2%)<br>No = 20 (12.9%)
To help others stop smoking in the future	Yes = 7 (4.5%)<br>No = 31 (20.1%)	Yes = 7 (4.5%)<br>No = 35 (22.6%)
**Satisfaction Ratings (1 = very unsatisfied; 5 = very satisfied), (M = Mean, SD = Standard Deviation)**		
**Question**	**Intervention (n = 42)**	**Control (n = 37)**
1. Information provided	3.8 (0.62)	3.9 (0.77)
2. Explanation of random allocation	3.6 (0.66)	3.6 (0.92)
3. Number of questionnaires	3.9 (0.67)	4.0 (0.73)
4. Length of questionnaires	3.9 (0.71)	4.1 (0.71)
5. Ease of completing questionnaires	4.0 (0.73)	4.2 (0.68)
6. Use of email to send questionnaires	4.1 (0.68)	4.2 (0.91)
7. Additional follow-up by the research team	3.5 (0.80)	3.5 (0.92)
8. Overall experience of taking part	3.9 (0.73)	3.8 (0.86)
**Future Intentions (1 = very unlikely; 5 = very likely)**		
9. Recommend taking part	3.7 (0.79)	3.5 (0.86)
10. Take part in a similar study	4.0 (0.64)	3.8 (0.85)
